# Supplementary material for: Pharmacogenomic Analysis Reveals CCNA2 as a Predictive Biomarker of Sensitivity to Polo-Like Kinase I Inhibitor in Gastric Cancer
Source: Cancers (Basel). 2020 May 30;12(6):1418. doi: 10.3390/cancers12061418 (PMC7352331; doi:10.3390/cancers12061418)
Supplement: Supplementary file 1 [file cancers-12-01418-s001.zip › cancers-824200 - supplementary.docx]

Supplementary Files

Pharmacogenomic Analysis Reveals *CCNA2* as a Predictive Biomarker of Sensitivity to Polo-Like Kinase I Inhibitor in Gastric Cancer

Yunji Lee, Chae Eun Lee, Sejin Oh, Hakhyun Kim, Jooyoung Lee, Sang Bum Kim and Hyun Seok Kim


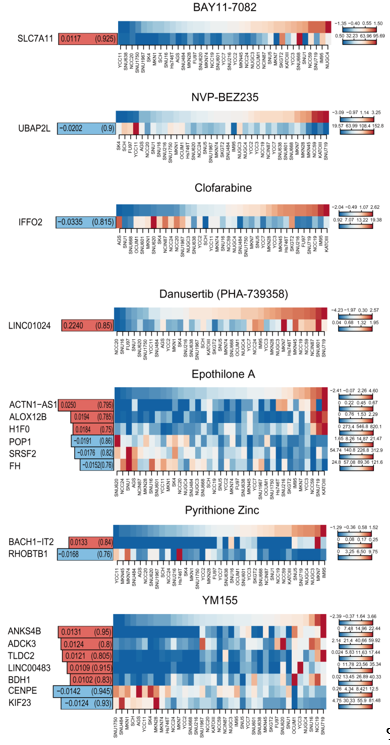


**Figure S1.** Identified biomarker and drug response relationships by the elastic net regularization method. The average weights of features are displayed in bar plots, and their frequencies are shown in parenthesis. Heat maps are depicted on a blue-white-red gradient scale of median-centered AUC values and expression levels (FPKM) of genes, respectively. Bar plots on the left are colored in red when the expression level of a biomarker is positively correlated with resistance of the given drugs and colored in blue when negatively correlated with drug resistance.


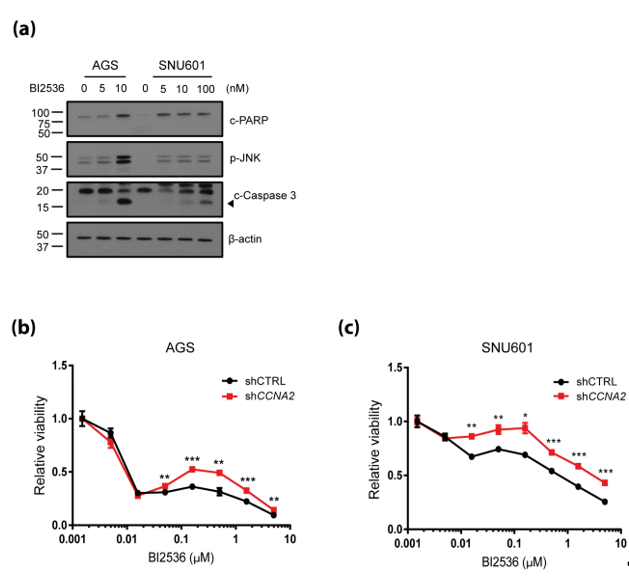


**Figure S2.** BI-2536 sensitivity in AGS and SNU601 cells. (**a**) Induction of apoptotic markers was assessed by immunoblotting. Whole cell lysates were prepared at 72 h post-BI-2536 or vehicle (DMSO) treatment with the indicated concentrations. (**b–c**) Dose-response curves of AGS and SNU601 cells expressing shCCNA2 or control shRNA after 72 h of exposure to BI-2536 at the indicated concentrations. **p* < 0.05, ***p* < 0.01, ****p* < 0.001; one-way ANOVA.


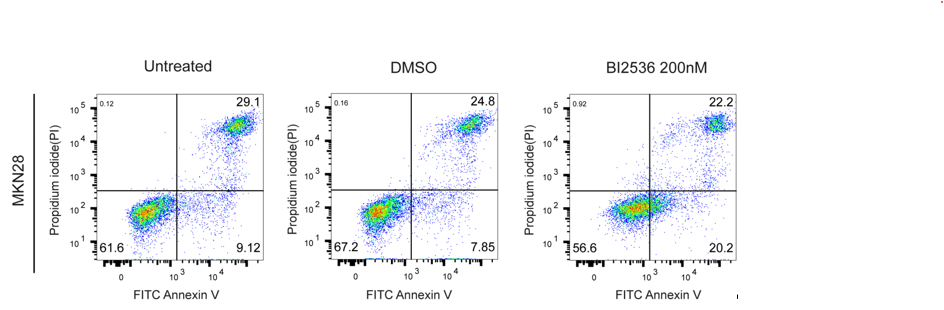


**Figure S3.** Apoptosis analysis using Annexin V-FITC/PI dual staining and flow cytometric analysis after BI-2536 (200 nM) treatment for 24 h are presented in scatter plots. Data points in the lower right quadrant represent early apoptotic cells.


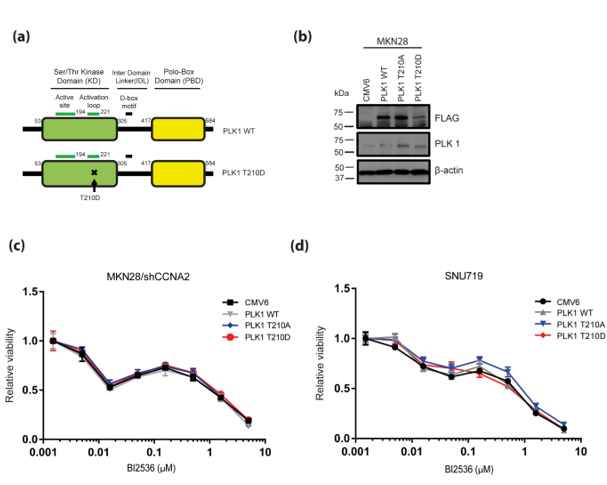


**Figure S4.** BI-2536 sensitivity with wild-type and mutant PLK1 levels. (**a**) Domain structure of wild-type and mutant PLK1. (**b**) Overexpression of flag-tagged wild-type (WT), non-phosphorylatable mutant (T210A), and phosphomimetic mutant (T201D) PLK1 in MKN 28 cells was visualized by Western blotting. (**c–d**) Dose-response curves of (**c**) MKN28/shCCNA2 cells and (**d**) SNU719 cells overexpressing empty plasmid (CMV6), wild-type, or mutant PLK1 3 days post BI-2536 treatment.

| 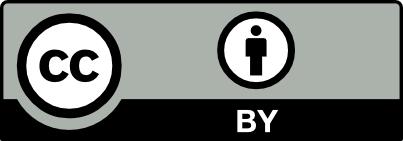 | © 2020 by the authors. Licensee MDPI, Basel, Switzerland. This article is an open access article distributed under the terms and conditions of the Creative Commons Attribution (CC BY) license (http://creativecommons.org/licenses/by/4.0/). |
| --- | --- |
