# Supplementary material for: Pharmacogenomic Analysis Reveals CCNA2 as a Predictive Biomarker of Sensitivity to Polo-Like Kinase I Inhibitor in Gastric Cancer
Source: Cancers (Basel). 2020 May 30;12(6):1418. doi: 10.3390/cancers12061418 (PMC7352331; doi:10.3390/cancers12061418)
Supplement: Supplementary file 1 [file cancers-12-01418-s001.zip › cancers-824200-Western Blots.pdf]

Fig. 2a

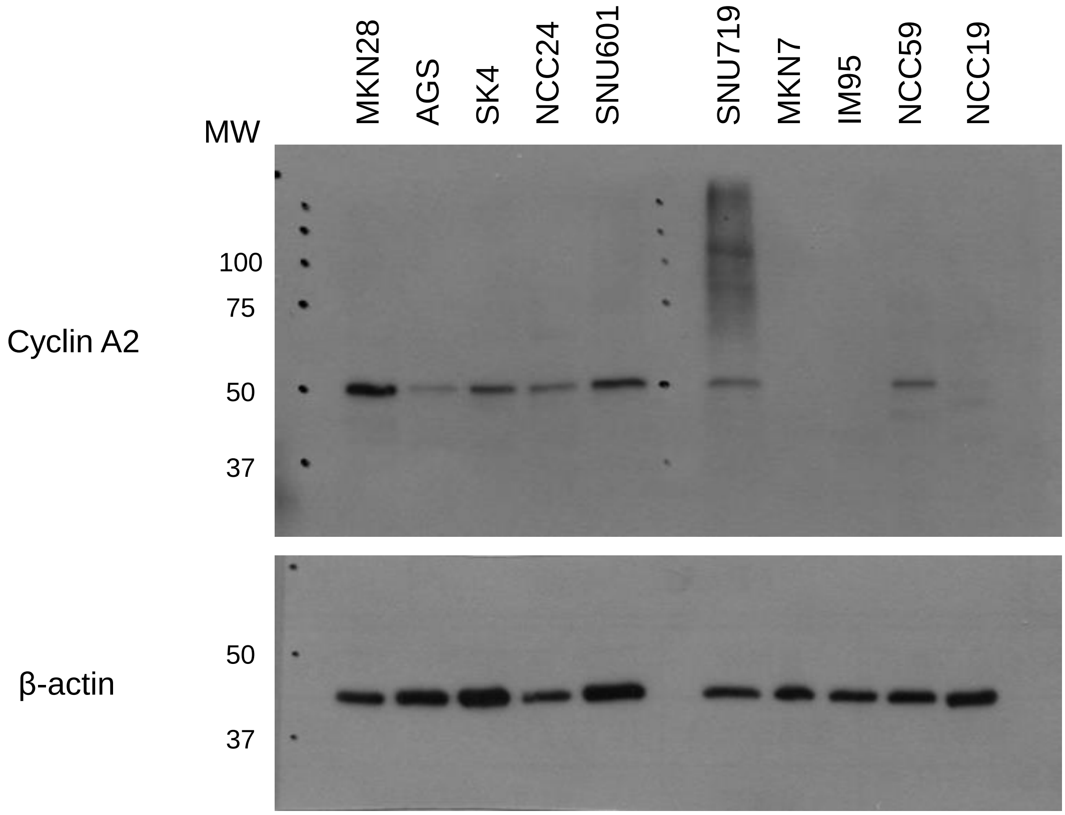

Band quantification was performed using ImageJ software

Figure 2 (a)	Normalized intensity (/β-actin)	Fold change
	Cyclin A2	
MKN28	1.03	1.00
AGS	0.15	0.14
SK4	0.31	0.31
NCC24	0.46	0.45
SNU601	0.40	0.39
SNU719	0.44	0.42
MKN7	0.04	0.04
IM95	0.11	0.11
NCC59	0.36	0.35
NCC19	0.10	0.10

Fig. 2c

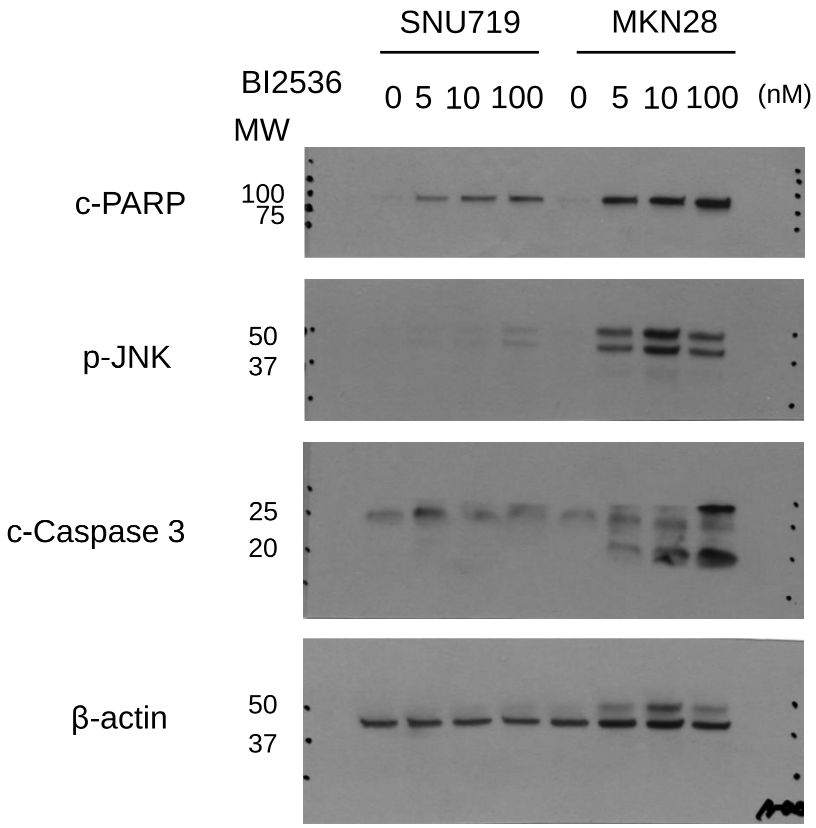

Figure 2 (c)	Normalized intensity (/β-acitn)			Fold change		
	c-PARP	p-JNK	c-Caspase 3	c-PARP	p-JNK	c-Caspase 3
SNU719 0nM	0.07	0.08	ND	1.00	1.00	ND
SNU719 5nM	0.31	0.13	ND	4.63	1.57	ND
SNU719 10nM	0.63	0.23	ND	9.40	2.84	ND
SNU719 100nM	0.72	0.47	ND	10.83	5.83	ND
MKN28 0nM	0.08	0.10	ND	1.21	1.22	ND
MKN28 5nM	0.77	0.98	0.22	11.49	12.14	1.00
MKN28 10nM	0.77	1.12	0.61	11.57	13.86	2.80
MKN28 100nM	1.22	1.05	1.06	18.30	13.00	4.85

* ND : Non-detectable

Fig. 2d

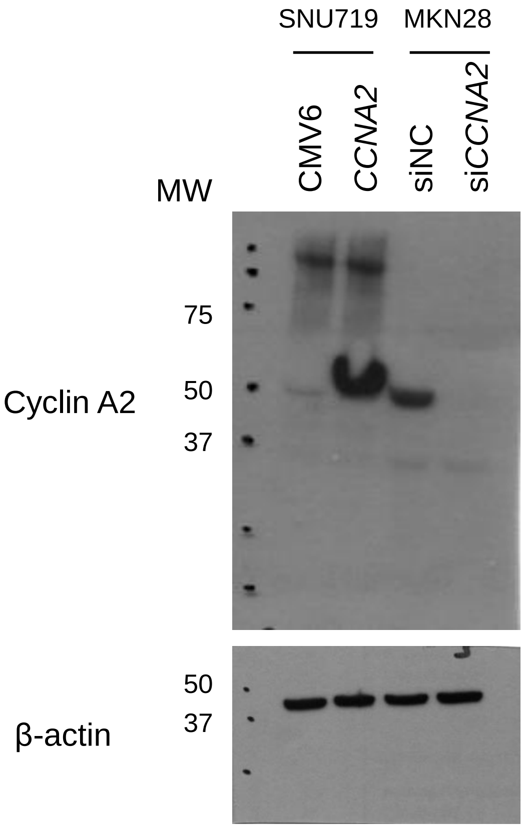

Figure 2 (d)	Normalized intensity (/β-actin)	Fold change
	Cyclin A2	Cyclin A2
SNU719 CMV6	0.11	1.00
SNU719 CCNA2	0.71	6.49
MKN28 siNC	0.29	1.00
MKN28 siCCNA2	0.02	0.06

Fig. 2g

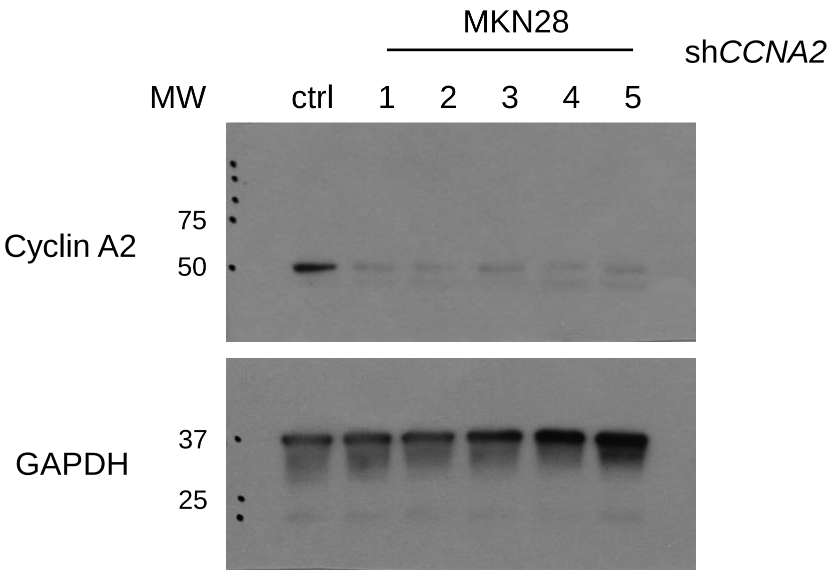

Figure 2 (g)	Normalized intensity (/GAPDH)	Fold change
	Cyclin A2	Cyclin A2
MKN28 CTRL	1.49	1.00
MKN28 #1	0.23	0.16
MKN28 #2	0.17	0.11
MKN28 #3	0.21	0.14
MKN28 #4	0.10	0.07
MKN28 #5	0.12	0.08

Fig. 3c

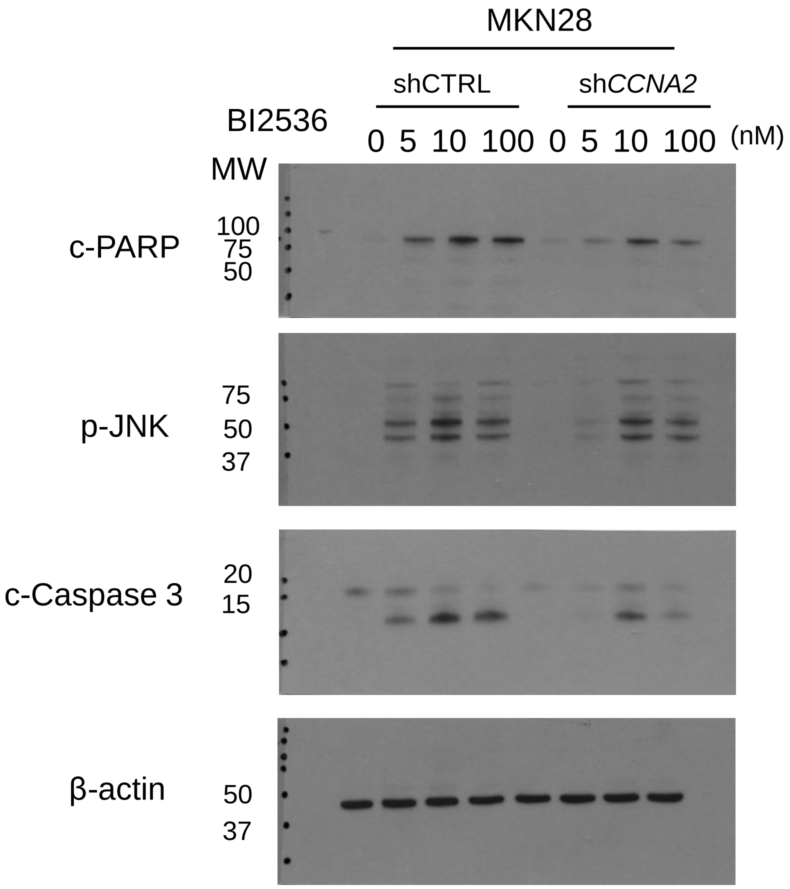

Figure 3 (c)	Normalized intensity (/β-actin)			Fold change		
	c-PARP	p-JNK	c-Caspase 3	c-PARP	p-JNK	c-Caspase 3
shCTRL 0nM	0.06	0.05	0.11	1.00	1.00	1.00
shCTRL 5nM	0.48	0.47	0.40	8.03	9.01	3.75
shCTRL 10nM	0.85	0.86	0.85	14.28	16.51	8.01
shCTRL 100nM	0.87	0.64	0.74	14.57	12.33	7.05
shCCNA2 0nM	0.17	0.16	0.09	2.90	2.98	0.87
shCCNA2 5nM	0.26	0.25	0.15	4.29	4.80	1.40
shCCNA2 10nM	0.61	0.72	0.56	10.28	13.91	5.32
shCCNA2 100nM	0.33	0.53	0.28	5.46	10.11	2.62

Fig. 4c

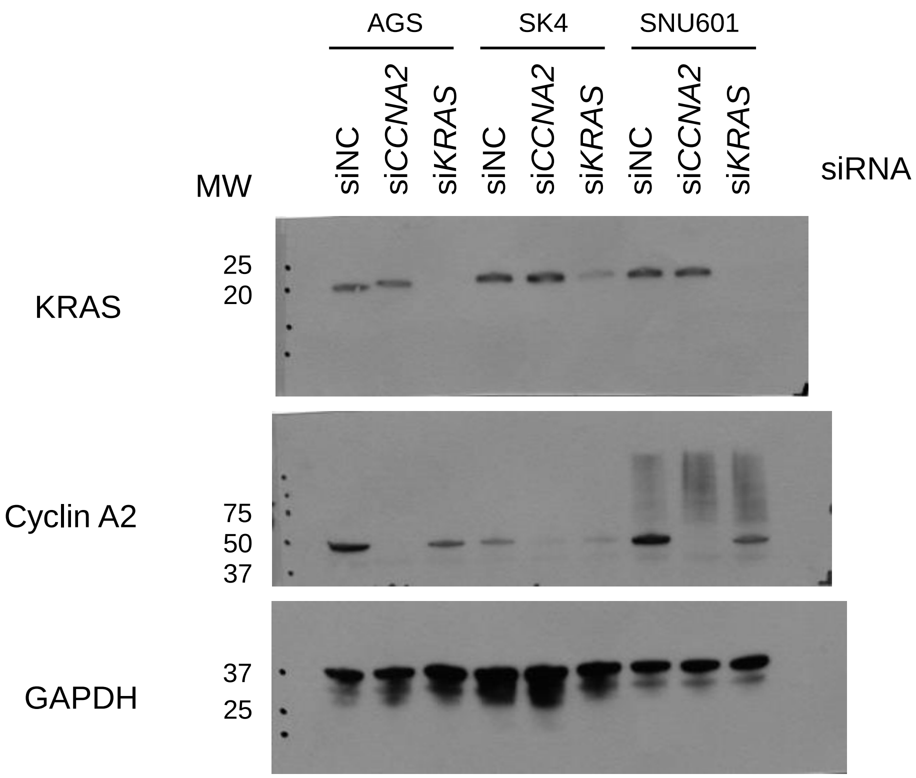

Figure 4 (c)	Normalized intensity (/GAPDH)		Fold change	
	KRAS	Cyclin A2	KRAS	Cyclin A2
AGS siNC	1.01	1.38	1.00	1.00
AGS siCCNA2	0.89	0.10	0.87	0.07
AGS siKRAS	0.10	0.39	0.10	0.28
SK4 siNC	0.71	0.22	1.00	1.00
SK4 siCCNA2	0.94	0.07	1.32	0.33
SK4 siKRAS	0.41	0.16	0.58	0.72
SNU601 siNC	1.24	1.32	1.00	1.00
SNU601 siCCNA2	1.05	0.29	0.85	0.22
SNU601 siKRAS	0.17	0.74	0.14	0.56

Fig. 4d

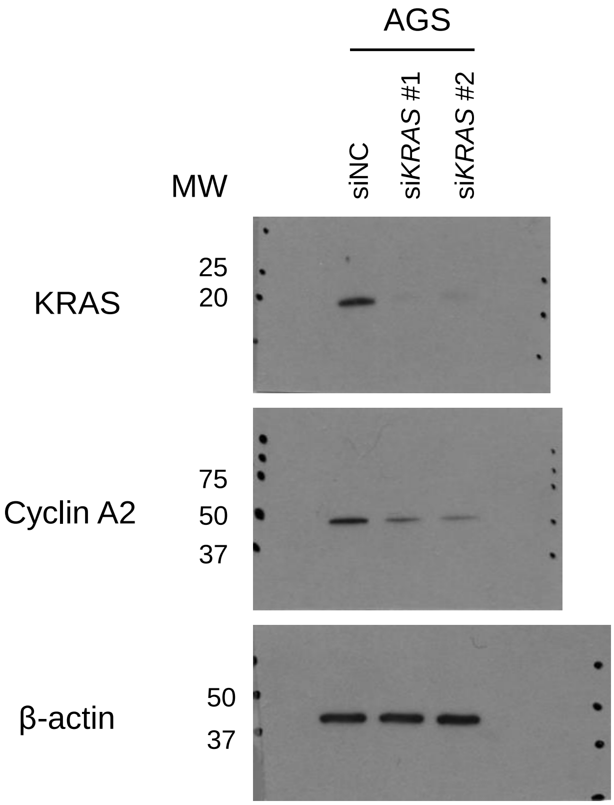

Figure 4 (d)	Normalized intensity (/β-actin)		Fold change	
	KRAS	Cyclin A2	KRAS	Cyclin A2
AGS siNC	0.98	0.95	1.00	1.00
AGS siKRAS #1	0.16	0.48	0.16	0.50
AGS siKRAS #2	0.24	0.24	0.25	0.25

Fig. 4e

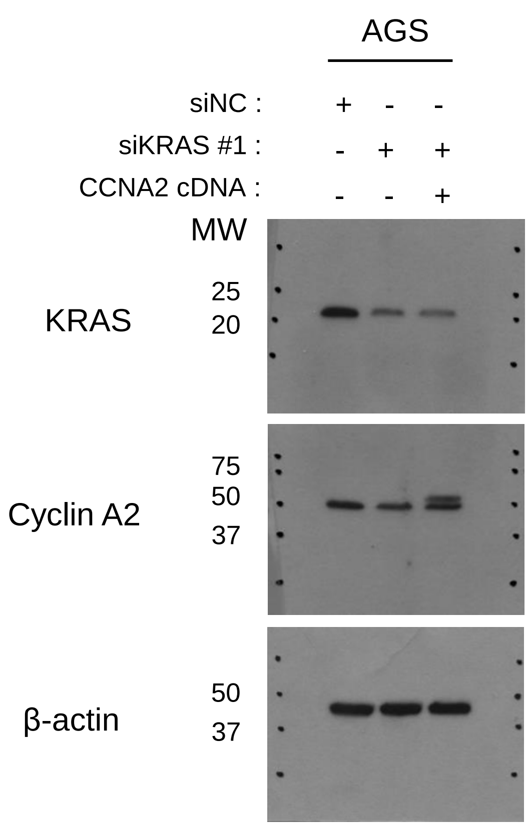

Figure 4 (e)	Normalized intensity (/β-actin)		Fold change	
	KRAS	Cyclin A2	KRAS	Cyclin A2
AGS siNC	1.07	0.63	1.00	1.00
AGS siKRAS	0.35	0.54	0.33	0.85
AGS siKRAS+CCNA2	0.33	0.95	0.30	1.51

Supple. 2a

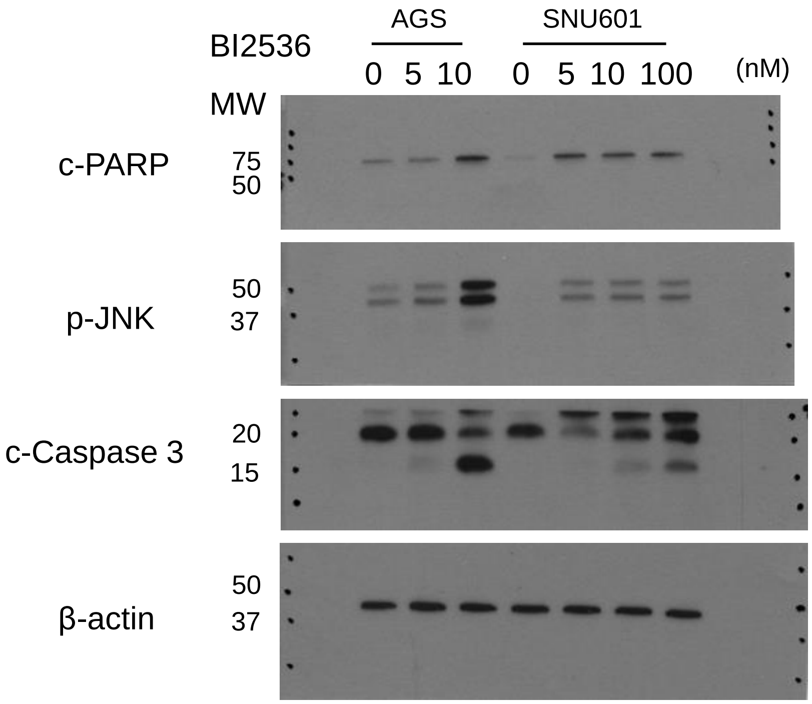

Supplementary Figure 2 (a)	Normalized intensity (/β-actin)			Fold change		
	c-PARP	p-JNK	c-Caspase 3	c-PARP	p-JNK	c-Caspase 3
AGS 0nM	0.32	0.27	0.06	1.00	1.00	1.00
AGS 5nM	0.30	0.34	0.16	0.95	1.26	2.84
AGS 10nM	0.91	1.04	1.03	2.83	3.90	18.00
SNU601 0nM	0.12	0.05	0.07	0.37	0.20	1.25
SNU601 5nM	0.67	0.27	0.10	2.08	1.02	1.78
SNU601 10nM	0.64	0.32	0.31	1.99	1.19	5.38
SNU601 100nM	0.60	0.31	0.54	1.87	1.17	9.43

Supple. 4b

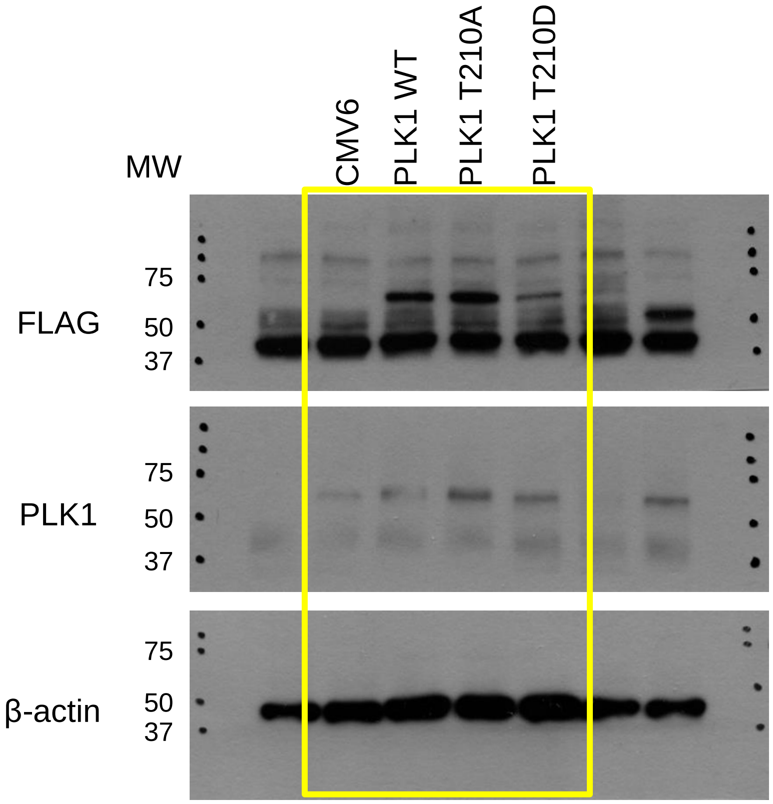

Supplementary Figure 4 (b)	Normalized intensity (/β-actin)		Fold change	
	FLAG	PLK1	FLAG	PLK1
CMV6	0.08	0.34	1	1
PLK1 WT	0.90	0.44	10.84	1.29
PLK1 T210A	1.04	0.92	12.59	2.72
PLK1 T210D	0.40	0.54	4.84	1.60
